# Identifying Unmet Needs of Informal Dementia Caregivers in Clinical Practice: User-Centered Development of a Digital Assessment Tool

**DOI:** 10.2196/59942

**Published:** 2025-04-07

**Authors:** Olga A Biernetzky, Jochen René Thyrian, Melanie Boekholt, Matthias Berndt, Wolfgang Hoffmann, Stefan J Teipel, Ingo Kilimann

**Affiliations:** 1 Deutsches Zentrum fuer Neurodegenerative Erkrankungen, Rostock/Greifswald, Gehlsheimer Strasse 20, Rostock, 18147, Germany, 49 381 494 9476; 2 Deutsches Zentrum fuer Neurodegenerative Erkrankungen, Rostock/Greifswald, Greifswald, Germany; 3 Institute for Community Medicine, Section Epidemiology and Community Health, University Medicine Greifswald, Greifswald, Germany; 4 Department for Psychosomatic and Psychotherapeutical Medicine, Rostock University Medical Center, Rostock, Germany

**Keywords:** unmet needs, assessment development, family caregivers of people with dementia, dementia, need, Alzheimer, self-guided, self-reported, caregiver, informal care, spousal care, interview, qualitative, thematic, usability, mHealth, tablet, self-completed, aging, patient care, health interventions, care giver, digital health, ehealth, digital assessment, memory

## Abstract

**Background:**

Despite the increasing interventions to support family caregivers of people with dementia, service planning and delivery is still not effective.

**Objective:**

Our study aimed to develop a digitally-supported needs assessment tool for family caregivers of people with dementia that is feasible, time-efficient, understood by users, and can be self-completed in the primary care setting.

**Methods:**

The development of the unmet needs assessment tool was part of a cluster-randomized controlled trial examining the effectiveness of a digitally supported care management programme to reduce unmet needs of family caregivers of people with dementia (GAIN [Gesund Angehörige Pflegen]) and was conducted in 3 phases. Using an iterative participatory approach with informal caregivers, health care professionals including general practitioners, neurologists, psychologists, psychiatrists, nurses, and Alzheimer Society representatives, we developed a digital self-completion unmet needs assessment tool focusing on informal caregivers’ biopsychosocial health und quality of life in connection to their caregiver responsibilities. Data were collected through group discussions, written feedback, protocols, think-aloud protocols, and interviews, and analyzed thematically.

**Results:**

Data from 27 caregivers, including caregivers of people with dementia (n=18), health care professionals (n=7), and Alzheimer Society representatives (n=2) were collected. Thematic analysis identified 2 main themes: content of the assessment tool and usability and handling of the digital tablet-based assessment tool. The feedback provided by the stakeholders led to new aspects and changes to make the tool comprehensive, easy to read, and easy to handle. The overall mean completion time was reduced from the initial 37 minutes to 18 minutes, which renders the assessment tool fit to be self-completed in waiting rooms of primary care practices or other settings.

**Conclusions:**

The input of the 3 stakeholder groups has supported the development of the assessment tool ensuring that all aspects considered important were covered and understood and the completion of the assessment procedure was time-efficient and practically feasible. Further validation of the assessment tool will be performed with the data generated as part of the GAIN trial.

## Introduction

The World Health Organization (WHO) estimates 55 million people are living with dementia worldwide, with an increase to 78 million by 2030 [1]. In Germany, approximately two-thirds of the 1.8 million people with dementia are cared for at home by an informal caregiver, most often a relative [2]. Numerous studies have shown that caring for a relative with dementia is associated with a multitude of time- and resource-intensive challenges [3-5]. Emotional stress, social isolation, depression, and financial burden are among the negative consequences that dementia family caregivers have reported [6-10]. To alleviate these consequences, interventions for the reduction of caregivers’ burden have been developed [11-14], such as telephone-based as well as multicomponent psychosocial and individualized support programs [11,13]. With the growing consensus that caregiver support is an integral part of the care of patients with dementia, there was also an increase in interventions to support informal caregivers [12]. However, each caregiver’s situation is different and requires an individual set of interventions. Individualized interventions are still underused in care practice as they require time and dementia-specific expertise that is often lacking in primary care. A systematic and comprehensive yet individualised unmet needs assessment tool would be useful for health care professionals (HCPs) to better plan services and interventions in clinical settings as it would flag areas where the individual caregiver needs support.

In a mixed methods study, Stirling et al [15] used Bradshaw taxonomy of need [16] to explore the relationship between different types of caregiver service need. Bradshaw and Care [16] defined categories of need as measures of professionally identified carer burden (normative need), service use (expressed need), carer’s stated need (felt need) and the comparison of groups using services with groups who do not (comparative need). It is argued that no single measure of the above needs is likely to capture all carer’s unmet needs. In the light of person-centred care, the normative need has been increasingly challenged as paternalistic and inappropriate [15,17].

Therefore, a focus on caregivers’ stated need (felt need) as well as service use (expressed need) would be an appropriate starting point to consider for an unmet needs assessment tool. An individualized, digitally supported self-assessment instrument can aid in the efficient identification of health and support areas that should be addressed from the perspective of the informal caregivers. This allows collaborative intervention planning and reduces the risk of a paternalistic doctor-patient relationship [18]. If a digital system is to truly ensure collaborative service planning, the digital assessments instrument must be developed with the participation of the users themselves.

The direct input of family caregivers and other stakeholders into the content, structure, and handling of the digital assessment system provides crucial feedback regarding its content validity as well as comprehensiveness and technical usability. Through a greater level of involvement of stakeholders, health care services can be improved and be more applicable in real-world clinical settings and facilitate implementation [19]. A systematic review of instruments used to assess the needs of family dementia caregivers identified 36 instruments that were described in detail or in part in the 70 publications included [20]. The authors reported that only one instrument was partially validated to assess the needs of family dementia caregivers, namely, the Carer’s Needs Assessment for Dementia (CNA-D), a semistructured interview not intended for clinical use with a completion time of about an hour. For conceptual clarity, researchers recommended establishing a theoretical model or framework to organize the diverse needs of family dementia caregivers [21]. In addition, researchers argue that a focus on the detection of changes in family caregivers’ needs throughout disease progression is important [20,22].

Therefore, the aim of this study was to develop a digital unmet needs assessment tool that can be used in primary care settings. We conducted the study in collaboration with informal and formal caregivers and other stakeholders. Our objectives were to (1) create a user-friendly and family dementia caregiver specific digitally supported assessment tool, (2) develop an assessment process that is understandable and comprehensive yet time-efficient, and (3) create an assessment tool that can be used at multiple locations such as general practitioners’ (GPs) offices and memory clinic waiting rooms.

## Methods

### Overview

The development of the assessment tool was in preparation of the upcoming randomized controlled intervention trial examining the effectiveness of a digitally supported care management intervention for family caregivers (German title: Gesund Angehörige Pflegen [GAIN]). The trial was supported by the Federal Joint Committee (G-BA [Gemeinsamer Bundesausschuss/Innovationsausschuss]). The funding code (FKZ) is 01VSF18030).

### Participants

We involved individuals from 3 stakeholder groups, namely, family dementia caregivers, health care professionals (general practitioners, neurologists, psychiatrists, psychologists, and nurses), and Alzheimer Society representatives. We approached HCPs of a medical university and memory clinic staff, as well as Alzheimer Society representatives, and asked for their feedback (phase 1). Family dementia caregivers who were visiting a memory clinic with their relative or friend with dementia were asked whether they would like to provide feedback on the paper-based version of the assessment instrument regarding the content, clarity, and comprehensiveness (phase 2) as well as the usability of the digital version of the assessment tool as a self-completion assessment instrument on a PC tablet (phase 3). No further demographic data were collected.

### Procedure

We used an iterative user-centered participatory approach with 3 phases. Previously used questionnaires [20,23,24] assessing unmet needs and care interventions from previous dementia care management studies [4,25-31] in Germany were used as starting point.

#### Phase 1

In phase 1, the authors developed a list of previously used questionnaires assessing unmet needs in family dementia caregivers as well as a list of available care interventions offered in Germany. The selection of questionnaires was based on a comprehensive review of the literature covering the databases OVID, MEDLINE, and PsycInfo searching for unmet needs, caregiver needs assessments methods and family dementia caregivers. With respect to available care interventions, we used work from previous dementia care management studies management studies [4,25-31] offering a list of interventions to family dementia caregivers in Germany. We used Germany-based intervention studies as these are health care system specific. Criteria for the inclusion of questionnaires were the content they covered (eg, quality of life, psychosocial factors, and health-related domains), their previous use in research and interventions, and their completion time. We also considered questionnaires based on either their recommendation by the EU Joint Programme in Neurodegenerative Research (JPND) Working Group on Longitudinal Cohorts or common use in larger German trials such as IDemUck [32], DelpHi-MV (Dementia: life- and person-centered help in Mecklenburg-Western Pomerania) [26,28], intersec-CM [33,34], or DemNet-D [35,36]. These questionnaires are validated and allowed comparison with German and international studies. The authors then further discussed these selections in meetings, compared 5 separate versions of a possible unmet needs assessment instrument and checked whether items would cover interventions offered as part of the dementia care management conducted in previous primary care studies [4,26,28,37,38]. With this approach, we wanted to ensure that the operationalization of problem-centered needs were connected to available services within the German health care system and could therefore be addressed in this framework accordingly.

The authors selected different assessment versions aiming to cover all biopsychosocial aspects and compared different scales for caregiver burden. The need items were then compared and matched with the list of informal caregiver interventions from previous studies—these were marked according to a match or no match. This allowed the calculation of percentages of “items covered” from the dementia caregiver intervention list for each version of questionnaires. The five versions of combinations of questionnaires were checked by 3 authors and researchers for plausibility. The results of this item-intervention matching process were used to identify which versions covered most unmet needs with matched possible interventions. These are shown in Table 1.

**Table 1.** Proportion of match with family dementia caregiver interventions.

Versions and interventions		Match, %
**Version 1**		47
	FIMA^a^	
	URN^b^	
	EQ-5D-5L^c^	
	ZBI-7^d^	
	LSNS-6^e^	
**Version 2**		17
	FIMA	
	URN	
	EQ-5D-5L	
	BIZA-D^f^	
**Version 3**		96
	CANE^g^	
	EQ-5D-5L	
	ZBI-7	
	LSNS-6	
**Version 4**		94
	CANE	
	DQoL-OC^h^	
**Version 5**		39
	HABC^i^	
	DQoL-OC	
	ZBI-7	

a FIMA: Questionnaire for the Use of Medical and Non-Medical Services in Old Age [39].

b URN: Caregiver unmet resource needs scale [40].

c EQ-5D-5L: Health-related quality of life [41].

d ZBI-7: Zarit Burden Interview [42,43].

e LSNS-6: Lubben Social Network Scale [44,45].

f BIZA-D: The Berlin inventory of the burden on relatives - dementia – Module 3,5,and 6 [46].

g CANE: Camberwell Assessment of Need for the Elderly [47,48].

h DQoL-OC: The Dementia Quality of Life Scale for Older Family Carers [49].

i HABC: Healthy Aging Brain Care Monitor—Caregiver Version [50].

Versions 3 and 4 covered most items, whereas versions 1, 2, and 5 covered less than 50% of the dementia-specific items. The two versions covering most unmet needs were then presented and discussed at an advisory board meeting with input from HCPs (eg, neurologists, psychiatrists, psychologists, and nurses) and Alzheimer Society representatives. Meeting minutes were recorded of all points raised and discussed. This input was used to select the final version of the assessment tool.

#### Phase 2

In phase 2, the chosen assessment version covering most unmet needs was tested in a memory clinic. A tablet computer was deemed most appropriate to serve as digital device due to its size, weight, flexibility, and ease of handling. Based on a previous study on the use of a tablet-based digital expert system [26,28,30], we had taken several decisions on the hardware basis of our system before the participatory part of our study started. The content and usability aspects, however, were developed together with future users. The authors approached family dementia caregivers in a memory clinic to provide feedback and suggestions on a paper-based version of the assessment tool. The changes were primarily focused on the Camberwell Assessment of Need for the Elderly (CANE) questionnaire, as well as the demographic and informal caregiver-specific questions, as these were items that could still be adapted. Family caregivers who were interested in contributing were given a short version comprising 7 questions of the CANE questionnaire. The researcher noted the caregivers’ comments and thoughts in a think-aloud protocol. Together with a researcher, they were asked to read the questions and share their thoughts with respect to each question and their understanding thereof. The focus was on comprehension, words and sentence structure, answer options, descriptions, and additional comments. In the second round, both informal caregivers and health care professionals were approached to provide feedback on the whole printed version of the assessment instrument. Those who agreed to provide feedback were asked to mock-complete the assessment tool and write down their notes and questions. In this round, informal caregivers and HCPs were asked to measure the time needed to complete the assessment procedure. We provided written questions such as “Are the questions clear and easy to understand?”; “Where did you encounter problems when completing the assessment?”; “Did you have issues understanding the questions?”; and “Are there questions or areas that should be in the assessment set but are currently missing?” Based on the feedback received, the first draft of the assessment set was modified resulting in a second draft. When adapting the first draft, our focus was on the assessment procedure being clear and easy to understand for family caregivers in terms of the way questions are phrased. Based on the feedback we received, several parts of the assessment set were adapted and duplicates were removed by the authors resulting in the final draft.

#### Phase 3

In phase 3, a digitalized tablet-based version of the final version of the assessment tool was tested for its technical usability. In this phase, we asked both informal caregivers and HCPs to test the digital assessment tool with a focus on the handling of the tablet. Data was collected through interviews and think-aloud protocols. Family caregivers in a memory clinic were asked to complete the digital assessment tool in the waiting room and share their thoughts and comments with a study nurse or researcher who protocolled the comments. Similarly, HCPs of a medical university and a memory clinic were asked to complete the digital assessment procedure and write down feedback on the handling and any possible bugs or problems they noticed while testing the digital system.

### Analysis

All data collected through written feedback, protocols, think-aloud protocols, and interviews were analyzed thematically following the steps by Braun and Clarke [51]. The six phases involved were (1) familiarizing ourselves with the data, (2) generating initial codes, (3) constructing themes, (4) reviewing themes, (5) defining and naming themes, and (6) producing the report [51].

### Ethical Considerations

The study is conducted in accordance with the criteria (valid at present) of the Declaration of Helsinki, the ICH-guidelines for Good Clinical Practice, the Memorandum for Safeguarding Good Scientific Practice (German Research Foundation), and the International Ethical Guidelines for Biomedical Research Involving Human Subjects. Ethical approval has been obtained from the Ethical Committee of the University Medicine Greifswald (BB120/2019) and the Ethical Committee of the University Medicine Rostock (A2020/0013). The trial is registered at ClinicalTrials.gov (NCT04037501).

## Results

Results of the development of the assessment tool of the 3 phases are divided in the subsections presented below. The process of phases 2 and 3 was iterative comprising several rounds through which we collected feedback, made changes based on feedback received and then moved to the next round of collecting feedback.

### Participants

A total of 27 family caregivers of people with dementia (n=18), HCPs (n=7), and Alzheimer Society representatives (n=2) participated. Among the HCPs were general practitioners, neurologists, psychologists, psychiatrists, and nurses. The mean age of the 27 family caregivers was 57 (SD 13.78; range 35‐88) years and 9 (50%) were women. The HCPs’ and representatives’ mean age was 41 (SD 8.05; range 28‐50) and 6 out of 9 (67%) were women. As this was a participatory approach, no further demographic data were collected.

### Phase 1: Structure and Content of the Assessment Tool

After the input from the advisory board including neurologists, psychiatrists, psychologists, nurses, and Alzheimer Society representatives, the final structure of the assessment instrument was selected. It was the one with the highest percentage of matches between problem-centered needs and the associated interventions (69/72, 96%; version 3; see Table 1).

The unmet needs assessment tool comprised several validated questionnaires with a total of 57 questions examining a person’s self-reported demographic information, use of medical and nonmedical services, unmet needs (CANE), health-related quality of life (EQ-5D-5L), caregiver burden (Zarit Burden Interview [ZBI]), and social support (Lubben Social Network Scale [LSNS]). It can be completed using a digital version or a paper-based version. In total, the assessment set covers 51 health- and support-related aspects.

Domains covered by the unmet needs assessment tool are the following: (1) health and care, (2) employment, (3) information and knowledge, (4) emotional support, (5) social support, and (6) caregiver burden.

The structure of the unmet needs assessment tool can be seen in Table 2.

**Table 2.** Structure of the unmet needs assessment tool.

Instrument	Domain
Socio-demographic information	—^a^
Caregiver specific information	Individual situation of informal caregiver
CANE^b^	Unmet needs I—Information, physical, mental
EQ-5D-5L	Health-related quality of life
LSNS^c^	Unmet needs II—Social network
ZBI^d^	Unmet needs III—Caregiver burden

a Not available.

b CANE: Camberwell Assessment of Need for the Elderly.

c LSNS: Lubben Social Network Scale.

d ZBI: Zarit Burden Interview.

The number of unmet needs addressed the participants’ medical needs, home care needs, psychosocial needs, and needs connected to the caregiver role. This needs assessment instrument included selected parts of the CANE [47].

Health-related quality of life was assessed using the EQ-5D-5L [41]. This instrument comprises 5 dimensions: mobility, self-care, usual activities, pain or discomfort, and anxiety or depression. Each dimension has 5 levels varying from no problems to extreme problems. Each level corresponds to a 1-digit number that expresses the level selected for that dimension ranging from 1 to 5 with higher numbers indicating more severe problems. The digits for the 5 dimensions can be converted into an overall index by an not publicly accessible algorithm. The index describes the participant’s health-related quality of life. It also contains a vertical visual analogue scale (EQ VAS), with endpoints that are labelled “Best imaginable health state” and “Worst imaginable health state.” The VAS reflects the patient’s own judgement and can be used as a quantitative measure of health outcome.

Social support was assessed using LSNS-6 [44,45]. This scale is a self-report measure of social engagement including contact to and interaction with family and friends on a 6-item scale. Total scores range from 0 to 30 with an equally weighted sum of the 6 items. The family and friends subscales include questions regarding the number of friends and family one has regular contact with as well as their availability for help and support in private matters. High scores indicate strong social networks.

Informal caregiver burden was assessed using the 7-item version of the Zarit-Burden Interview (ZBI-7). The short version ZBI is a caregiver self-report measure to examine burden, which is associated with functional and behavioral impairments in the social, psychological and physiological context, and home care situation [42,43]. It contains 7 items using a 5-point scale. Response options range from 0 (Never) to 4 (Nearly to Always). Total scores range from 0 indicating no burden to 28 indicating severe burden.

### Phases 2 and 3: Feedback Received on the Assessment Tool

#### Themes

A total of two main themes were derived from the analysis: (1) content of the assessment instrument and (2) digital tablet usability for the assessment tool. Each theme had further subthemes and subtheme categories and were divided in either informal caregivers or HCPs and Alzheimer Society representatives as these groups had different areas they paid special attention to. Almost all subthemes had 1 to 3 subtheme categories, see Table 3.

**Table 3.** Themes, subthemes, and subtheme categories.

Theme and subtheme	Subtheme category
1. Content of the assessment procedure	—^a^
Informal caregivers	—
1.1 Comprehension	1.1.1 Simplification of questions 1.1.2 Simplification of answer options 1.1.3 Separate questions for caregiver health and caregiver care responsibilities in relation to the person with dementia
1.2 Assessment structure	1.2.1 No multiple questions in a table 1.2.2 Questions whether help is needed in a specific domain for clear identification of unmet needs
1.3 Reduction of completion time	1.3.1 Fatigue
1.4 Areas important to caregivers	—
Health care professionals	
1.1 Assessment structure	1.1.1 Order of instruments 1.1.2 Focus on caregivers’ health and care responsibilities
1.2 Reduction of completion time	—
2. Digital tablet usability of the assessment tool	—
Informal caregivers	
2.1 Simple layout	2.1.1 Large font and buttons
2.2 Handling	2.2.1 Manual answer selection
Health care professionals	
2.1 Simple layout	2.1.1 One question per screen 2.1.2 Option to go back and forth within the assessment procedure 2.1.3 Progression bar
2.2 Handling	—

a Not applicable.

##### Content of the Assessment Instrument

Regarding the assessment’s content, informal caregivers provided information on 4 subthemes regarding the comprehension, assessment structure, reduction of completion time, and areas important to them.

###### Comprehension (Informal Caregiver Perspective)

Regarding the clarity of the assessment procedure to them, caregivers commented on the simplification of questions and answer options. Instead of “This is an unmet need” or “This is a need met” (as suggested in the CANE) they preferred to answer “Yes” or “No,” for instance. They also commented on being sometimes confused whether a question was referring to themselves or to the person diagnosed with dementia indicating that questions had to be framed specifically.

###### Assessment Structure

Regarding the assessment’s structure, informal caregivers commented that multiple questions in a table format were confusing. In addition, some commented that they would like to self-identify the areas where they needed help rather than having professionals assume that they needed help or support in an area, which was incorporated into the assessment procedure by first asking whether there is a problem in a specific area and secondly asking whether the respondent receives enough help with respect to this problem. A yes or no response to these questions would then indicate the presence or absence of an unmet need.

HCPs and Alzheimer Society representatives considered the order of instruments within the assessment procedure and argued that the instrument covering most unmet needs (CANE) should be moved to the front to prevent that important health care domains received less attention due to fatigue. They also reported that the informal caregivers’ own health as well as factors influencing their health due to their caregiver role should be a focus since a caregiver’s unmet needs can arise from two sources: either from the personal needs of the family caregiver or from his or her care responsibilities for the people with dementia.

###### Reduction of Completion Time (Informal Caregiver Perspective)

The majority of the informal caregivers commented on the length of the initial versions of the assessment tool and reported that they became a bit tired towards the end of the assessment procedure. A caregiver stated: “For the last few questions my focus was a bit lost” (informal caregiver, woman).

###### Areas Important to Caregivers (Informal Caregiver Perspective)

Caregivers commented on some of the areas that they found particularly important such as psychological support, mobility, social life, and psychoeducation.

A caregiver stated:

*I would say that there should be a question for psychological support. Self-help groups should be offered locally and it should be asked whether advice or support is needed in this regard. Another thing that I would still find important is to ask how mobile you are and whether you need help in this regard. For example, I don’t have a car and have to use public transport to get everywhere. And there are also people who don’t even have a driver’s license. And if you have to go somewhere and have to pick up things from the pharmacy (eg, incontinence pads), these are sometimes large packages that are also heavy and you have to get them home first. It would be a good idea to ask whether you need help in this regard*.

[Informal caregiver, woman]

Another caregiver reported:

*The social life is very important, my father is hardly involved in it anymore. Because of his swallowing problems, he can no longer even go to a restaurant, which is difficult.*

[Informal caregiver, man]

Another cargiver stated:

*Since we have not yet come into contact with services, I would like a lot of information so that you know what you have to do. And also so that you know why behavior changes, how people then think and what is going on inside you. I’d be very interested in that.*

[Informal caregiver, man]

Regarding the content of the assessment instrument, HCPs and Alzheimer Society representatives provided information on 2 subthemes, namely, assessment structure and reduction of completion time.

### Digital Tablet Usability of the Assessment Tool

Regarding the assessment’s tablet usability, informal caregivers, HCPs, and Alzheimer Society representatives identified 2 subthemes, namely, simple layout and handling with slightly different subtheme categories.

#### Simple Layout

Informal caregivers commented on the font size, often taking out their reading glasses to be able to read the questions.

HCPs commented that one question per screen would allow the large font and button size needed. Some clinicians stated that it would be helpful to allow caregivers to go back within the assessment procedure in case they changed their mind regarding a previous answer. Going forward should only be possible if the question on the screen was completed. The majority of HCPs stated that an indication bar within the assessment procedure would be helpful for caregivers to receive information on their progress as well as how much of the assessment procedure is still in front of them.

#### Handling

The majority of informal caregivers reported that selecting answers by clicking worked well after adjustments were made to the font size and the size of the answer option buttons. Regarding the handling, all HCPs and Alzheimer Society representatives agreed that the selection of answers should be as straight forward as possible allowing caregivers to complete the assessment procedure without technical issues.

**Figure 1.**
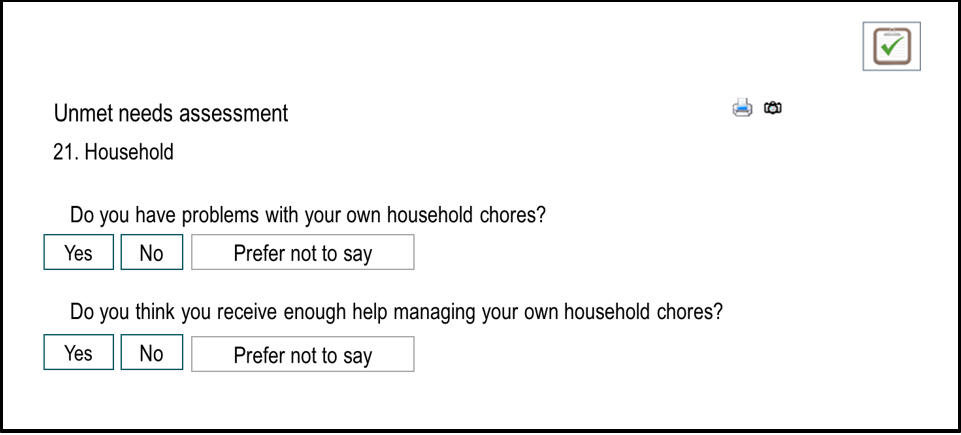
Outline of the first digital assessment version.

**Figure 2.**
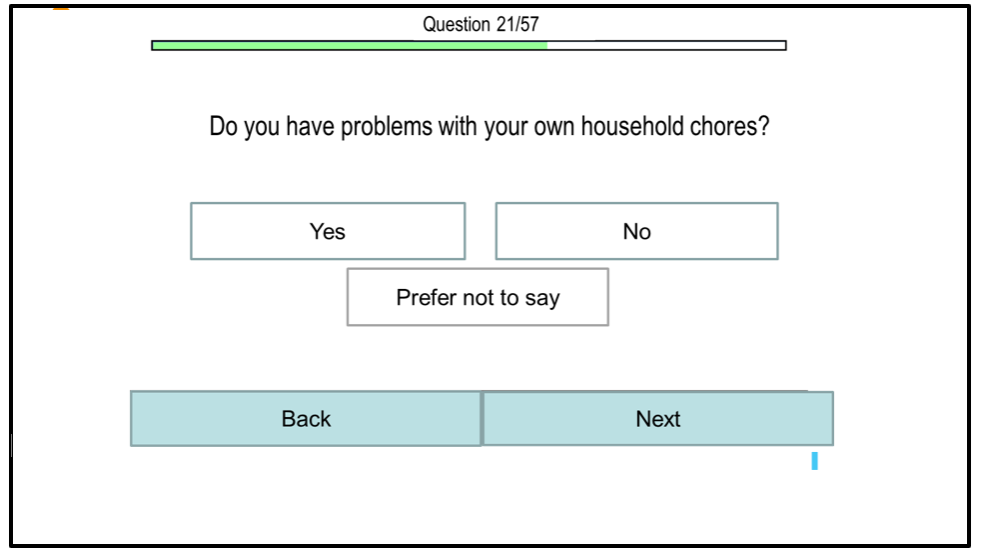
Outline of the final digital assessment version.

The adaptations made to the digital tablet-based assessment tool are shown in Figures1  2. Figure 1 shows the initial, first version of the tablet-based assessment tool. Figure 2 shows the final tablet-based assessment version after the feedback was incorporated into the development by the IT team.

After completion, the outcome of the assessment procedure can be viewed by the study nurse as well as the GP. These professionals can then use the outcome to work together with the informal caregiver toward a reduction of the unmet needs identified. The study nurses provide the informal caregivers with a summary of the consultation where points discussed are listed.

**Figure 3.**
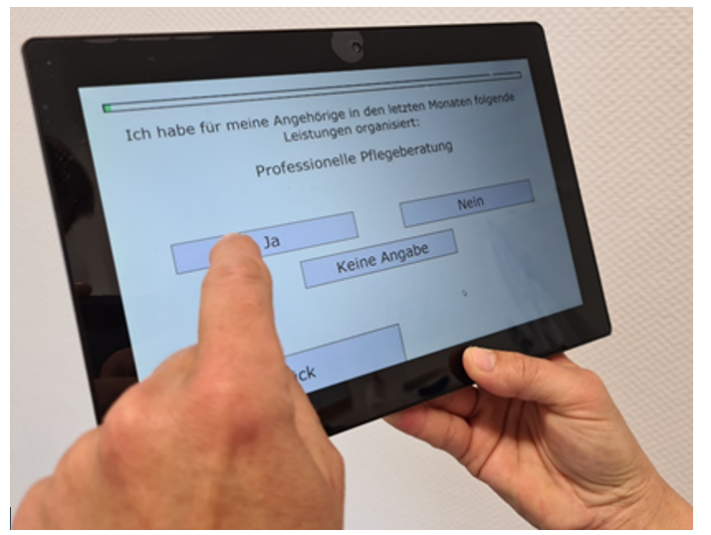
An informal caregiver completing the digital tablet-based assessment.

Figure 3 depicts a caregiver filling out the digital version of the assessment tool on a tablet.

## Discussion

### Principal Findings

This study describes the phases of an iterative, participatory process we used to develop a digital tablet-based assessment tool designed to comprehensively identify the needs of informal caregivers of people with dementia in clinical practice. Through the collaboration with family dementia caregivers, health care professionals, and Alzheimer Society representatives, we developed a digital unmet needs assessment instrument that consists of a set of standardized instruments but also includes additional questions and adaptations that were incorporated along the iterative development process. The unmet needs assessment tool focuses on needs that can arise from two sources: either from the personal needs of the family caregiver or from the care responsibilities for people with dementia. With the contribution of informal caregivers and other stakeholders, we designed an assessment tool that covers a comprehensive range of needs considered important. At the same time, with the input of the stakeholders, the time to complete the assessment procedure was substantially reduced from the initial 37 minutes to 18 minutes. This indicates that we reached our goal of developing a comprehensive, feasible, and time-efficient assessment device.

With respect to the content of the assessment instrument, we identified important aspects regarding clarity, assessment structure, the reduction of completion time as well as content areas important to informal caregivers. We took the feedback in multiple rounds and incorporated changes along the way. Similar approaches have been conducted successfully by other research teams developing assistive technologies to support self-management of people with dementia [52].

Regarding the digital tablet-based assessment tool, we could incorporate crucial aspects regarding the layout and the handling of the tablet. The difference between our first digital assessment version compared with the final digital assessment version illustrates the importance of involving multiple perspectives, especially of those for whom the assessment instrument or technology is intended. In the literature, there is an increase of studies involving patients and stakeholders in the development or testing of technology [52-54].

The participatory approach allowed us to incorporate aspects into the development of the digital assessment tool that we, as researchers, would have missed otherwise, as can be seen in the first digital tablet-based assessment version in Figure 1 when compared with the final version in Figure 2. The input of informal caregivers, health care professionals, and Alzheimer Society representatives has broadened our perspective and has given us important hints and advice towards aspects that are important for a digital, self-completion unmet needs assessment tool. They identified questions that were unclear, suggested possible answer options, and gave us ideas on how to simplify the navigation throughout the assessment procedure. Some researchers have used similar procedures for the development of instruments to assess patients’ needs [24,47] or to facilitate implementation [19]. A review by Fischer et al [55] reports on the importance of user involvement, particularly the involvement of older adults in technology design. In their review of 40 empirical studies published between 2014 and 2018, these authors examined the consequences of involving older adults in technology design and stated that learning, adjusted design, and improved sense of participation were outcomes frequently stated in studies [55]. Not only did they report that involvement facilitated learning about the needs of older adults but it also led to the generation of new technology ideas such as a companion robot, for instance, which was not in the researchers’ mind before involving older adults [55]. Newly gained insights then lead to iterative adjustments of the prototype design [55]. This was also the process that we followed making adjustments to the unmet needs assessment tool. With respect to the quality of technology developed with user involvement, Kopeć and et al [56] noticed that the overall quality of the technology could be improved. They argued that teams who involved users to develop a mobile app were rated higher by an independent jury in a competition than teams who did not. Older adults appreciated the sense of participation and felt that they were being treated as experts on their own lives and equal partners [57]. Researchers reported that involved users enjoyed their experience and some users described their involvement as “happy memories” [58-60].

In contrast to already existing unmet needs assessment instruments, our digital assessment tool identifies a informal caregiver’s self-reported unmet needs and ties them directly to interventions most of which are offered within the German health care system. Through the matching process of possible unmet needs with interventions in phase 1 of our assessment development process, we aimed to overcome the pitfalls of identifying problem-centered needs without having support services to meet them at hand. In addition, the digital assessment tool acknowledges the intertwined nature of the dyad, namely the informal caregiver and the person with dementia focusing on not only the health and support needs of the caregiver but also the caregiver’s care responsibilities. Although we did not include the CNA-D, we did compare the content and ensured that no important aspect was missed in the assessment procedure.

### Limitations

The stakeholders who contributed to the development of the assessment tool were a convenience sample. We have asked informal caregivers, health care professionals and Alzheimer Society representatives who either visited a memory clinic with their significant other, worked at the memory clinic or who were already known to us due to previous collaborations. Furthermore, the sample is limited to people living in a specific region in Northern Germany (Mecklenburg-Western Pomerania). In addition, our level of involvement was limited to stakeholders sharing their feedback on the assessment tool, which is only 1 of the 3 levels of involvement [61]. The tendency to involve stakeholders only in some rather than all levels of involvement was also highlighted by Fischer et al [55] arguing for further research on the actual practices of user involvement. For future projects, a higher level of involvement is sought, especially since current results indicate that an elaborated, theory-based participatory approach including people with dementia, informal caregivers and regional stakeholders might raise the chances of successful implementation in routine care [19].

### Conclusion

Our study has shown the feasibility and success of user involvement in the development of an assessment tool for identifying the unmet needs of people with dementia and their informal caregivers. By presenting our approach, we hope to motivate researchers worldwide to expand stakeholder involvement in the early stages of studies. To facilitate this, the questionnaire will be made available to researchers on the homepage of the Network for Translational Dementia Care Research. Further evaluations, such as the acceptance of the tool from the perspective of the study nurses and GPs, are currently being evaluated and will be published separately.

## Abbreviations

CANE: Camberwell Assessment of Need for the Elderly
CNA-D: Carer’s Needs Assessment for Dementia
DelpHi-MV: Dementia: life- and person-centered help in Mecklenburg-Western Pomerania
G-BA: Gemeinsamer Bundesausschuss/Innovationsausschuss
GAIN: Gesund Angehörige Pflegen
GP: general practitioner
HCP: health care professional
JPND: EU Joint Programme in Neurodegenerative Research
LSNS: Lubben Social Network Scale
VAS: visual analog scale
WHO: World Health Organization
ZBI: Zarit Burden Interview

## References

[1] (2021). Dementia. World Health Organization.

[2] Blotenberg I, Hoffmann W, Thyrian JR (2023). Dementia in Germany: epidemiology and prevention potential. Dtsch Arztebl Int.

[3] Schulz R, Martire LM (2004). Family caregiving of persons with dementia: prevalence, health effects, and support strategies. Am J Geriatr Psychiatry.

[4] Zwingmann I, Hoffmann W, Michalowsky B (2018). Supporting family dementia caregivers: testing the efficacy of dementia care management on multifaceted caregivers’ burden. Aging Ment Health.

[5] Michalowsky B, Thyrian JR, Eichler T (2016). Economic analysis of formal care, informal care, and productivity losses in primary care patients who screened positive for dementia in Germany. J Alzheimers Dis.

[6] Murray J, Schneider J, Banerjee S, Mann A (1999). EUROCARE: A cross-national study of co-resident spouse carers for people with Alzheimer’s disease: II—A qualitative analysis of the experience of caregiving. Int J Geriat Psychiatry.

[7] Marin D, Amaya K, Casciano R (2003). Impact of rivastigmine on costs and on time spent in caregiving for families of patients with Alzheimer’s disease. Int Psychogeriatr.

[8] Baiyewu O, Smith-Gamble V, Akinbiyi A (2003). Behavioral and caregiver reaction of dementia as measured by the neuropsychiatric inventory in Nigerian community residents. Int Psychogeriatr.

[9] Schneider J, Murray J, Banerjee S, Mann A (1999). EUROCARE: a cross-national study of co-resident spouse carers for people with Alzheimer’s disease: I--Factors associated with carer burden. Int J Geriatr Psychiatry.

[10] Brodaty H, Donkin M (2009). Family caregivers of people with dementia. Dialogues Clin Neurosci.

[11] Berwig M, Heinrich S, Spahlholz J, Hallensleben N, Brähler E, Gertz HJ (2017). Individualized support for informal caregivers of people with dementia – effectiveness of the German adaptation of REACH II. BMC Geriatr.

[12] Williams F, Moghaddam N, Ramsden S, De Boos D (2019). Interventions for reducing levels of burden amongst informal carers of persons with dementia in the community. A systematic review and meta-analysis of randomised controlled trials. Aging Ment Health.

[13] Shata ZN, Amin MR, El-Kady HM, Abu-Nazel MW (2017). Efficacy of A multi-component psychosocial intervention program for caregivers of persons living with neurocognitive disorders, Alexandria, Egypt: A randomized controlled trial. Avicenna J Med.

[14] Lök N, Bademli K (2017). Pilot testing of the “First You Should Get Stronger” program among caregivers of older adults with dementia. Arch Gerontol Geriatr.

[15] Stirling C, Andrews S, Croft T, Vickers J, Turner P, Robinson A (2010). Measuring dementia carers’ unmet need for services - an exploratory mixed method study. BMC Health Serv Res.

[16] Bradshaw J, Care M (1972). Medical Care: Essays on Current Research Seventh Series.

[17] Meaney AM, Croke M, Kirby M (2005). Needs assessment in dementia. Int J Geriatr Psychiatry.

[18] Asadi-Lari M, Tamburini M, Gray D (2004). Patients’ needs, satisfaction, and health related quality of life: towards a comprehensive model. Health Qual Life Outcomes.

[19] Seidel K, Quasdorf T, Haberstroh J, Thyrian JR (2022). Adapting a dementia care management intervention for regional implementation: a theory-based participatory barrier analysis. Int J Environ Res Public Health.

[20] Novais T, Dauphinot V, Krolak-Salmon P, Mouchoux C (2017). How to explore the needs of informal caregivers of individuals with cognitive impairment in Alzheimer’s disease or related diseases? A systematic review of quantitative and qualitative studies. BMC Geriatr.

[21] Kipfer S, Pihet S (2020). Reliability, validity and relevance of needs assessment instruments for informal dementia caregivers: a psychometric systematic review. JBI Evid Synth.

[22] Bressan V, Visintini C, Palese A (2020). What do family caregivers of people with dementia need? A mixed-method systematic review. Health Soc Care Community.

[23] Mansfield E, Boyes AW, Bryant J, Sanson-Fisher R (2017). Quantifying the unmet needs of caregivers of people with dementia: a critical review of the quality of measures. Int J Geriatr Psychiatry.

[24] Wancata J, Krautgartner M, Berner J (2005). The Carers’ Needs Assessment for Dementia (CNA-D): development, validity and reliability. Int Psychogeriatr.

[25] Dreier-Wolfgramm A, Michalowsky B, Austrom MG (2017). Dementia care management in primary care: Current collaborative care models and the case for interprofessional education. Z Gerontol Geriatr.

[26] Thyrian JR, Hertel J, Wucherer D (2017). Effectiveness and safety of dementia care management in primary care: a randomized clinical trial. JAMA Psychiatry.

[27] Zwingmann I, Michalowsky B, Esser A (2019). Identifying unmet needs of family dementia caregivers: results of the baseline assessment of a cluster-randomized controlled intervention trial. J Alzheimers Dis.

[28] Thyrian JR, Fiß T, Dreier A (2012). Life- and person-centred help in Mecklenburg-Western Pomerania, Germany (DelpHi): study protocol for a randomised controlled trial. Trials.

[29] Dreier A, Thyrian JR, Eichler T, Hoffmann W (2016). Qualifications for nurses for the care of patients with dementia and support to their caregivers: A pilot evaluation of the dementia care management curriculum. Nurse Educ Today.

[30] Thyrian JR, Winter P, Eichler T (2017). Relatives’ burden of caring for people screened positive for dementia in primary care : Results of the DelpHi study. Z Gerontol Geriatr.

[31] Eichler T, Thyrian JR, Hertel J (2016). Unmet needs of community-dwelling primary care patients with dementia in Germany: prevalence and correlates. J Alzheimers Dis.

[32] Köhler L, Meinke-Franze C, Hein J (2014). Does an interdisciplinary network improve dementia care? Results from the IDemUck-study. Curr Alzheimer Res.

[33] Nikelski A, Keller A, Schumacher-Schönert F (2019). Supporting elderly people with cognitive impairment during and after hospital stays with intersectoral care management: study protocol for a randomized controlled trial. Trials.

[34] Schumacher-Schönert F, Boekholt M, Nikelski A (2024). Versorgungslücken nach dem Krankenhausaufenthalt schließen: Studienergebnisse [intersec-CM] zum Entlass- und Überleitungsmanagement nach § 39 SGB V für Menschen, die im Krankenhaus kognitive demenzielle Beeinträchtigungen zeigen [in German]. Zeitschrift für Evidenz, Fortbildung und Qualität im Gesundheitswesen.

[35] Gräske J, Meyer S, Schmidt A (2016). Regional dementia care networks in Germany--results from the DemNet-D-Study regarding the quality of life of their users. Pflege.

[36] Laporte Uribe F, Wolf-Ostermann K, Wübbeler M, Holle B (2018). Care arrangements in dementia care networks: findings from the DemNet-D study baseline and 1-year follow-up. J Aging Health.

[37] Zülke A, Luck T, Pabst A (2019). AgeWell.de - study protocol of a pragmatic multi-center cluster-randomized controlled prevention trial against cognitive decline in older primary care patients. BMC Geriatr.

[38] Thyrian JR, Eichler T, Pooch A (2016). Systematic, early identification of dementia and dementia care management are highly appreciated by general physicians in primary care - results within a cluster-randomized-controlled trial (DelpHi). J Multidiscip Healthc.

[39] Seidl H, Bowles D, Bock JO (2015). FIMA – Fragebogen zur Erhebung von Gesundheitsleistungen im Alter: entwicklung und pilotstudie [Article in German]. Gesundheitswesen.

[40] Higginson IJ, Gao W, Jackson D, Murray J, Harding R (2010). Short-form Zarit Caregiver Burden Interviews were valid in advanced conditions. J Clin Epidemiol.

[41] Lubben J, Blozik E, Gillmann G (2006). Performance of an abbreviated version of the Lubben Social Network Scale among three European community-dwelling older adult populations. Gerontologist.

[42] Zank S, Schacke C, Leipold B (2006). Berliner Inventar zur Angehörigenbelastung - Demenz (BIZA-D). Z Klin Psychol Psychother.

[43] Reynolds T, Thornicroft G, Abas M (2000). Camberwell Assessment of Need for the Elderly (CANE). development, validity and reliability. Br J Psychiatry.

[44] Oliveira DC, Vass C, Aubeeluck A (2018). The development and validation of the Dementia Quality of Life Scale for Older Family Carers (DQoL-OC). Aging Ment Health.

[45] Monahan PO, Alder CA, Khan BA, Stump T, Boustani MA (2014). The Healthy Aging Brain Care (HABC) Monitor: validation of the Patient Self-Report Version of the clinical tool designed to measure and monitor cognitive, functional, and psychological health. Clin Interv Aging.

[46] Stein J, Pabst A, Weyerer S (2016). The assessment of met and unmet care needs in the oldest old with and without depression using the Camberwell Assessment of Need for the Elderly (CANE): Results of the AgeMooDe study. J Affect Disord.

[47] King RB, Hartke RJ, Lee J, Raad J (2013). The stroke caregiver unmet resource needs scale: development and psychometric testing. J Neurosci Nurs.

[48] EuroQol Group (1990). EuroQol - a new facility for the measurement of health-related quality of life. Health Policy.

[49] Gray J, Kim J, Ciesla JR, Yao P (2016). Rasch analysis of the Lubben Social Network Scale-6 (LSNS-6). J Appl Gerontol.

[50] Hébert R, Bravo G, Préville M (2000). Reliability, validity and reference values of the Zarit Burden Interview for assessing informal caregivers of community-dwelling older persons with dementia. Can J Aging.

[51] Braun V, Clarke V (2006). Using thematic analysis in psychology. Qual Res Psychol.

[52] Øksnebjerg L, Woods B, Ruth K (2020). A tablet app supporting self-management for people with dementia: explorative study of adoption and use patterns. JMIR Mhealth Uhealth.

[53] Hettinga M, De Boer J, Goldberg E, Moelaert F (2009). Navigation for people with mild dementia. Stud Health Technol Inform.

[54] van de Vijver S, Hummel D, van Dijk AH (2022). Evaluation of a digital self-management platform for patients with chronic illness in primary care: qualitative study of stakeholders’ perspectives. JMIR Form Res.

[55] Fischer B, Peine A, Östlund B (2020). The importance of user involvement: a systematic review of involving older users in technology design. Gerontologist.

[56] Kopeć W, Balcerzak B, Nielek R, Kowalik G, Wierzbicki A, Casati F (2018). Older adults and hackathons: a qualitative study. Empir Software Eng.

[57] Maaß S, Buchmüller S (2018). The crucial role of cultural probes in participatory design for and with older adults. i-com.

[58] Eftring H, Frennert S (2016). Designing a social and assistive robot for seniors. Z Gerontol Geriatr.

[59] Lee HR, Šabanović S, Chang WL Steps toward participatory design of social robots: mutual learning with older adults with depression.

[60] Alaoui M, Ting KLH, Lewkowicz M (2014). The urge for empirically-informed design of social-oriented AAL applications – the example of 2 AAL projects. Procedia Comput Sci.

[61] Arnstein SR (1969). A ladder of citizen participation. J Am Inst Plann.

